# Functionality, Anthropometric Measurements, and Handgrip Strength in Community-Dwelling Older Adults

**DOI:** 10.3390/healthcare14010030

**Published:** 2025-12-22

**Authors:** Daiane Pereira Santos, Claudinéia Matos de Araújo Gesteira, Claudio Henrique Meira Mascarenhas, Helen Cristiny Tedoro Couto Ribeiro, Tatiane Dias Casimiro Valença, Elaine dos Santos Santana, Luciana Araújo dos Reis

**Affiliations:** 1 Health Department 1, Southwestern Bahia State University, Jequié 45200-000, Bahia, Brazil; 202010565@uesb.edu.br (D.P.S.); claudineia.matos@uesb.edu.br (C.M.d.A.G.); claudio.henrique@uesb.edu.br (C.H.M.M.); tatianedias@uesb.edu.br (T.D.C.V.); 2 Nursing Department, Federal University of São João del-Rei, Divinopóles 35500-001, Minas Gerais, Brazil; helen.cristiny@ufsj.edu.br; 3 Health Sciences Research Unit: Nursing, Coimbra School of Nursing, 36550-000 Coimbra, Portugal; elainesantana@esenfc.pt

**Keywords:** aging, daily activities, hand strength, functional capacity, anthropometry

## Abstract

**Highlights:**

**What are the main findings?**

- A higher body mass index and abdominal adiposity were associated with reduced functional capacity for instrumental activities in community-dwelling older adults.
- Handgrip strength showed significant associations with anthropometric indicators, reinforcing its value as a quick and feasible screening tool in primary health care.

**What are the implications of the main findings?**

- Simple measures such as handgrip strength, BMI, and waist circumference can help identify older adults at nutritional and functional risk in routine primary health care.
- Early screening with these indicators can guide targeted interventions to prevent functional decline and promote healthy aging.

**Abstract:**

**Introduction**: Functionality, anthropometric measurements (BMI, arm circumference), and handgrip strength (HGS) are crucial for assessing the health of older adults, as HGS is a strong predictor of frailty and independence, correlating with muscle mass loss (sarcopenia) and the risk of falls. **Background/Objectives**: To analyze the relationship between functional capacity, anthropometric measurements, and handgrip strength in community-dwelling older adults. **Methods**: A descriptive, exploratory, cross-sectional study with a quantitative approach was conducted with 225 older adults monitored at two Family Health Units, using the Barthel Scale, Lawton and Brody Scale, anthropometric measurements (body mass index, waist, calf, and brachial circumferences), and dynamometry as instruments. Spearman’s test was used for correlations, with interpretation by shared variance and comparison of magnitudes by Steiger r-to-z method. A higher frequency of females (65.8%) was observed, in the age range between 60 and 68 years (51.1%), independent in Basic Activities of Daily Living (76.9%) and dependent in Instrumental Activities of Daily Living (99.1%). The analysis revealed that waist circumference showed a significant correlation with waist-to-hip ratio (ρ-value 0.604; *p*-value < 0.01) and body mass index (ρ-value = 0.696; *p*-value < 0.01). These associations showed shared variances of 36.5% (waist circumference and waist-to-hip ratio) and 48.4% (waist circumference and body mass index). Waist-to-hip ratio showed a significant positive correlation with waist-to-hip ratio (ρ-value = 0.256; *p*-value < 0.01) and body mass index (ρ-value = 0.198; *p*-value < 0.01). However, these relationships showed lower shared variances at 6.5% with waist-to-hip ratio and 3.9% with BMI. The Lawton scale showed a statistically significant negative correlation with hand grip strength (ρ-value = −0.176; *p*-value < 0.01). **Conclusions**: There is a significant relationship between functional capacity, anthropometric measurements, and hand grip strength in community-dwelling older adults, reflecting the interaction between physical performance, body composition, and autonomy.

## 1. Introduction

Human aging is a progressive process that involves anatomical, physiological, and psychosocial changes. These changes make older people more susceptible to disease, which can result in losses in functional capacity. As a consequence, difficulties arise in performing activities of daily living, which compromises quality of life and promotes physical and social vulnerability [1].

Among the changes resulting from this process, the reduction in muscle mass stands out, often accompanied by a decrease in strength and power. This decline directly compromises the mobility and functionality of older adults and is associated with muscular, neurological, endocrine, and environmental factors. These limitations impact the performance of essential activities, such as walking, sitting, and standing up [2].

In this context, handgrip strength (HGS) is an important functional indicator, as it is directly related to maintaining independence in older adults. A reduction in this strength can result from both muscle inactivity and motor neuron degeneration, which are common events in the aging process [3]. In addition, anthropometric measurements, such as body mass index (BMI) and waist and calf circumferences, are relevant parameters, as they allow the identification of risks associated with sarcopenia, obesity, and functional loss, providing essential information for clinical and nutritional interventions [4].

Considering the aging population, preserving the functionality of older adults is essential to promote physical well-being and reduce the burden on healthcare systems and caregivers. Understanding the relationship between functional capacity, anthropometric measurements, and muscle strength is therefore essential to support more effective therapeutic interventions. Handgrip strength (HGS), in addition to reflecting overall muscle condition, is associated with relevant clinical outcomes such as falls, frailty, and mortality. Thus, its analysis, when performed in conjunction with anthropometric and functional parameters, can guide both the development of more specific rehabilitation programs and the formulation of public policies aimed at the well-being of the older population. Given this, the present study aims to analyze the relationship between functional capacity, anthropometric measurements, and handgrip strength in older adults living in the community. And as a research question: what is the relationship between functional capacity, anthropometric measurements, and handgrip strength in older adults living in the community?

Functionality, anthropometric measurements (BMI, arm circumference), and handgrip strength (HGS) are crucial for assessing the health of older adults, as HGS is a strong predictor of frailty and independence, correlating with muscle mass loss (sarcopenia) and the risk of falls. It is influenced by age, sex, and nutritional status, and indicates the need for early intervention in older adults in the community to maintain autonomy and quality of life, through simple assessments such as dynamometer testing.

## 2. Materials and Methods

This is a descriptive, exploratory study with a cross-sectional design and a quantitative approach. Data from the research project entitled Construction of assistive/care technologies for dependent older adults and their family caregivers in primary care in Brazil and Portugal were analyzed. For the purposes of this study, only data relating to the municipality of Jequié/BA were analyzed.

The study was conducted in two Health Units in the municipality of Jequié/BA. This municipality has an estimated population of 158,812 inhabitants and is located in the interior of the State of Bahia, 365 km from the capital [5].

### 2.1. Participants, Instruments and Procedures

All individuals aged 60 or older, of both sexes, registered in the two health units were selected to participate in the study. Individuals who could not be located at their homes after three attempts, or those who, at the time of the interview, did not have an adequate informant present if they were unable to understand the instructions due to cognitive impairment, were excluded from the research.

Cognitive screening was performed with the elderly using the Mini-Mental State Examination (MMSE) by Folstein, Folstein, and McHugh (Cronbach’s reliability coefficient/alpha around 0.80), specifically, the version used in Brazil and adapted by Bertolucci et al. [6]. Therefore, a score of 17 or less represented unsatisfactory cognitive conditions.

In this context, the use of the instrument allowed for the screening of individuals with severe cognitive decline, to minimize the bias that could be caused by the low educational level of the respondents.

**Figure 1:**
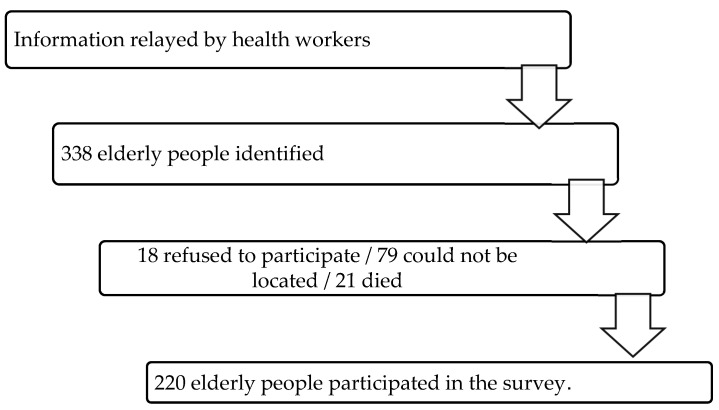
The sample selection process.

The sample selection process is schematically represented in Figure 1. This is a non-probabilistic convenience sampling, in which participants were selected according to availability and accessibility in the health units participating in the study, respecting the previously defined inclusion criteria.

The research instruments consisted of sociodemographic data (age group, sex, education, family income, and marital status), Barthel Scale (basic activities of daily living), Lawton and Brody Scale (instrumental activities of daily living), anthropometry (body mass index/BMI, waist circumference/WC, waist-to-hip ratio/WHR, and brachial circumference/BC), and dynamometry (muscle strength assessment).

The Barthel Scale (Reliability coefficients/Cronbach’s alpha greater than 0.80) [7] was used to assess basic activities of daily living (eating, bathing, dressing, personal hygiene, urination, defecation, use of the toilet, transfer between bed and chair, walking, and climbing stairs), with scores ranging from 0 to 100, where Independence: 100 points; mild dependence: 60–95; moderate dependence: 40–55; severe dependence: 20–35; and total dependence: less than 20.

The Lawton and Brody Scale (Reliability coefficients/Cronbach’s alpha of approximately 0.94) [8] was used to assess instrumental activities of daily living (use of the telephone, travel, shopping, meal preparation, housework, medication use, and finances). The score ranges from 0 to 21, with total dependence being less than or equal to 5; partial dependence being greater than 5 and less than 21; and independence being 21. For the present study, the sample was classified as independent: score = 21 and dependent: score < 21 points.

BMI was calculated as the body mass of older adults in kilograms (kg) divided by height squared, with the following categories: underweight (BMI ≤ 22); normal weight (BMI < 27); and overweight/obese (BMI ≥ 27) [9]. The participant’s body mass was measured using a portable digital platform scale with a maximum capacity of 150 kg and an accuracy of 100 g. Height was measured using a portable stadiometer, positioning the older person with their head in accordance with the Frankfurt plane [10].

Body circumferences were measured using a flexible, inelastic tape measure with a scale in millimeters. For each anatomical region, three consecutive measurements were taken, and the average was used for analysis. The abdominal waist circumference was measured at the midpoint between the last rib and the iliac crest, with the participant standing and without skin compression [10]. The calf circumference was measured with the participant seated, knee flexed at 90°, at the point of greatest circumference of the dominant leg. Additionally, the ankle circumference was measured immediately above the malleoli [11]. Finally, the brachial circumference was measured with the arm extended along the body, palm facing the thigh, and at the midpoint between the acromion and the olecranon [12].

Handgrip strength (HGS) was assessed using an electronic dynamometer (E.Clear, model EH101), following the protocol of the American Association of Hand Therapists [13]. For the test, the individual remained seated in a chair, with shoulders in a neutral position, one hand resting on the thigh, the elbow of the evaluated limb flexed at 90°, and the forearm in neutral rotation. Between attempts, an average interval of one minute was observed for recovery. Each participant was encouraged to apply maximum force, and the best score among three attempts was recorded for each hand. Individuals with MFP < 27 kgf (men) and <16 kgf (women) were classified as dynapenic [14]. The average of the 3 values relating to the dominant limb is evaluated.

The data was collected after authorization from the Municipal Health Department, the coordinators of the Family Health Units (USFs), and approval by the Research Ethics Committee. The collection was carried out between July 2022 and May 2024, conducted by the team from the Interdisciplinary Center for Studies and Research on Human Aging (NIEPEH), composed of undergraduate and graduate students.

### 2.2. Statistical Methods

Statistical analyses were performed using descriptive and inferential analysis in the SPSS Statistical Program version 22.0 (https://www.ibm.com/products/spss-statistics, accessed on 18 December 2025). Descriptive analyses included the calculation of absolute and relative frequencies for categorical variables and means and standard deviations for continuous variables.

The normality of the scores for functionality, handgrip strength, and anthropometric measurements was assessed using the Kolmogorov–Smirnov test. The results showed that the variables “FUNCTIONALITY,” “ANTHROPOMETRIC MEASUREMENTS,” and “HANDGRIP STRENGTH” did not have a normal distribution, with (K-S(225) = 0.412, *p* < 0.001; S-W(225) = 0.420, *p* < 0.001) and (K-S(225) = 0.091, *p* < 0.001; S-W(225) = 0.958, *p* < 0.001), respectively.

Spearman’s correlation test was adopted in this analysis due to the non-normal distribution of variables, as verified by preliminary normality tests. This nonparametric test is appropriate for measuring the strength and direction of the association between ordinal or quantitative variables that do not follow a normal distribution, based on data rankings rather than raw values. To interpret the effect size of the correlations, shared variance was used, calculated by squaring the correlation coefficient (r^2^) and then multiplying by 100, expressing the value as a percentage. This procedure indicates the proportion of the variance of one variable that can be explained by the other, providing an estimate of the magnitude of the association between the pairs of variables. Additionally, the comparison between correlation magnitudes was performed using Steiger r-to-z transformation. This method converts Spearman’s correlation coefficients into z-values, enabling statistical comparison between two correlation coefficients from the same sample. The test assesses whether the difference between the correlations is statistically significant, contributing to the identification of more robust associations.

The project was approved by the Ethics Committee of the Independent College of the Northeast (FAINOR), under CAAE number: 36278120.0.2002.5578 and approval protocol Opinion Number: 4.351.219.

## 3. Results

This study found a higher frequency of females (65.8%), with partners (51.1%), who can read and write (87.6%), aged between 60 and 68 years (51.1%), and with an income of two or more minimum wages (51.1%). In terms of health conditions, the largest distribution was of older adults with pain (68.9%), with more than one chronic disease (52.4%), who use medication (85.8%), independent in Basic Activities of Daily Living/BADL (76.9%), dependent in Instrumental Activities of Daily Living/IADL (99.1%), and overweight/obese (45.3%).

The results of the tests are presented in Table 1.

The values presented by the study participants in BMI (26.54 ± 5.02) and HGS (24.43 ± 8.76) are in line with the average values for older adult (BMI = Adequate weight/normal weight between 22.0 and 27.0 and HGS = between 21 and 22 kgf) [8,14]. A high average was also observed in ABVD (96.24 ± 10.25) and a low average in AIVD (18.10 ± 3.08).

Spearman’s correlation analysis revealed that WC had a significant positive correlation with WHR (ρ-value 0.604; *p*-value < 0.01) and BMI (ρ-value = 0.696; *p*-value < 0.01), suggesting that larger WC measurements are associated with greater overall and central body fat accumulation, characteristics related to increased BMI and waist-to-hip ratio. These associations showed shared variance of 36.5% (WC and WHR) and 48.4% (WC and BMI) (Table 1).

HGS showed a significant positive correlation with WHR (ρ-value = 0.256; *p*-value < 0.01), indicating that individuals with greater central fat accumulation and body mass index also have higher levels of muscle strength, which may reflect characteristics of total body mass, including lean mass.

The Lawton scale, which assesses instrumental functionality, showed a statistically significant negative correlation with BMI (ρ-value = −0.004; *p*-value < 0.01) and HGS (ρ-value = −0.176; *p*-value < 0.01), suggesting that higher BMI and strength values are associated with lower levels of instrumental functionality (Table 2).

## 4. Discussion

The functional assessment revealed significant independence in ADLs and greater dependence in IADLs. This pattern is expected in older populations, as IADLs require greater autonomy, planning, and judgment, making them more susceptible to early functional losses associated with chronic diseases, physiological decline, and cognitive changes [15,16,17].

The nutritional status assessed by BMI indicated that a significant proportion of participants were overweight or obese. Among older adults, excess weight, especially when combined with sarcopenia, is associated with increased frailty, reduced walking speed, metabolic syndrome, and an elevated risk of functional decline [18,19,20].

The HGS presented values slightly below the national reference average, although still within the expected limits for older adults. This difference may be related to the predominance of females and the high average age of the sample [21]. Values below 27 kgf for men and 16 kgf for women are widely used as cut-off points for identifying frailty, and their reduction is associated with an increased risk of falls, disability, and mortality, reinforcing their importance as a marker of functional vulnerability [22].

It was observed that increased BMI, especially when associated with abdominal obesity, was related to poorer functional performance, particularly in ADLs. Even in the presence of preserved muscle strength, excess visceral fat compromises mobility and impairs the performance of complex tasks, a result that is consistent with previous evidence [23]. In addition, WC showed a positive correlation with BMI and WHR, reinforcing the role of central adiposity as a marker of metabolic and functional risk in the older population [24].

The analysis also revealed a positive, albeit weak, correlation between HGS and anthropometric indicators such as BMI and WHR. This finding suggests that higher body mass values, when associated with greater lean mass, may favor muscle strength. However, the accumulation of abdominal fat tends to have the opposite effect, contributing to a decline in strength and functionality [25,26].

Among the limitations of the study, we highlight the cross-sectional design, as it does not allow causal inferences about the associations observed. In addition, we recognize that the inclusion of only older adults with preserved cognition and mobility may represent some selection bias, as it excludes more frail individuals. The analysis did not take into account potential confounding factors, such as physical activity level, eating habits, income, and educational level, which can simultaneously influence strength, functionality, and body composition. In this sense, it is recommended that longitudinal studies be conducted in the future to deepen the understanding of these relationships and guide more precise interventions aimed at maintaining functional capacity in older adults.

One limitation is the cross-sectional design, which makes it impossible to establish causal relationships between the variables analyzed. In addition, the exclusive inclusion of people with preserved cognition and mobility may have generated selection bias, since individuals in more fragile conditions were excluded from the sample.

Another limitation of the study was the lack of control for fundamental variables such as physical activity, nutritional status, musculoskeletal conditions, risk of falls, depression, income, and education.

## 5. Conclusions

It is concluded that although the correlations found in the present study between functional capacity, anthropometric measurements, and HGS in the elderly individuals evaluated were weak, there is evidence that adequate strength and body mass reflect in better functional capacity. Therefore, it is suggested that further studies be conducted to verify the existence or not of this correlation. Although greater strength and body mass suggest better functional reserve, an imbalance of these factors, such as abdominal obesity, can compromise functionality, especially in IADLs. The correlations found reinforce the use of HGS, associated with BMI and WC, as an early screening tool in primary care, as it is accessible and effective in identifying functional and nutritional risks. Based on this, integrated interventions with physical exercise, nutritional guidance, and therapeutic activities that preserve functionality and stimulate cognitive performance are recommended. These findings support public policies aimed at promoting autonomy and healthy aging, especially in vulnerable populations.

## Figures and Tables

**Table 1:** Results of handgrip strength, functionality, and anthropometric body composition tests.

Tests	Mean and SD±
Handgrip strength (kgf)	24.43 (±8.76)
Lawton and Brody Scale (IADL)	18.10 (±3.08)
Escala de Barthel (ADL)	96.24 (±10.25)
Age	71.05 (±8.31)
Weight (kg)	67.22 (±13.79)
Height (m)	1.58 (±0.10)
BMI (kg/m^2^)	26.54 (±5.02)
Waist circumference (cm)	92.98 (±13.07)
Waist-to-hip ratio	0.92 (±0.08)
Upper arm circumference	30.98 (±5.01)
Calf circumference	34.94 (±3.96)

Source: survey data.

**Table 2:** Spearman correlation analyses between anthropometric indicators of body composition, handgrip strength, and functionality in older adults.

	WHR	WC	Barthel	BMI	HGS	Lawton
WHR	-					
WC	0.604 **	-				
Barthel	0.001	−0.010	-			
BMI	0.197 **	0.696 **	0.015	-		
HGS	0.256 **	−0.088	0.191 **	0.063	-	
LAWTON	−0.098	−0.018	0.368 **	0.004 **	−0.176 **	-

Note: ** = *p*-value < 0.01. Source: survey data.

## Data Availability

The data supporting the findings of this study are not publicly available due to ethical and privacy restrictions but can be obtained from the corresponding author upon reasonable request.
